# Chromosome movements in meiotic prophase: regulatory mechanisms and biological roles

**DOI:** 10.3389/fcell.2026.1911284

**Published:** 2026-08-14

**Authors:** Li Zhao, Jinmin Gao

**Affiliations:** 1 Center for Cell Structure and Function, Key Laboratory of Animal Resistance Biology of Shandong Province, Collaborative Innovation Center of Cell Biology in Universities of Shandong, College of Life Sciences, Shandong Normal University, Jinan, China; 2 State Key Laboratory of Medicinal Chemical Biology, Cancer Biology Center, Tianjin Key Laboratory of Protein Science, College of Life Sciences, Nankai University, Tianjin, China

**Keywords:** chromosome motion, LINC complex, meiosis, nuclear envelope, pairing, synapsis, telomere

## Abstract

Meiotic prophase is characterized by dynamic chromosome movements that are essential for accurate genome transmission and the generation of genetic diversity. During this stage, chromosome ends, typically telomeres or other specialized chromosomal domains, attach to the nuclear envelope, where the conserved LINC complexes transmit cytoskeletal forces to drive coordinated chromosome motion. These movements are regulated by kinase-controlled chromosome end-nuclear envelope attachment, meiosis-specific LINC components, cytoskeletal motor networks, membrane fluidity, nuclear lamina dynamics, and other factors. Accumulating evidence indicates that chromosome movements play crucial roles in homolog pairing and synapsis, contribute to the resolution of chromosome entanglements, influence recombination and crossover patterning, and may also have post-synapsis functions. Here, we review the molecular machinery that drives meiotic chromosome movements, the mechanisms that regulate their dynamics, and their diverse biological functions during meiotic prophase.

## The major chromosomal challenges of meiosis

**FIGURE 1:**
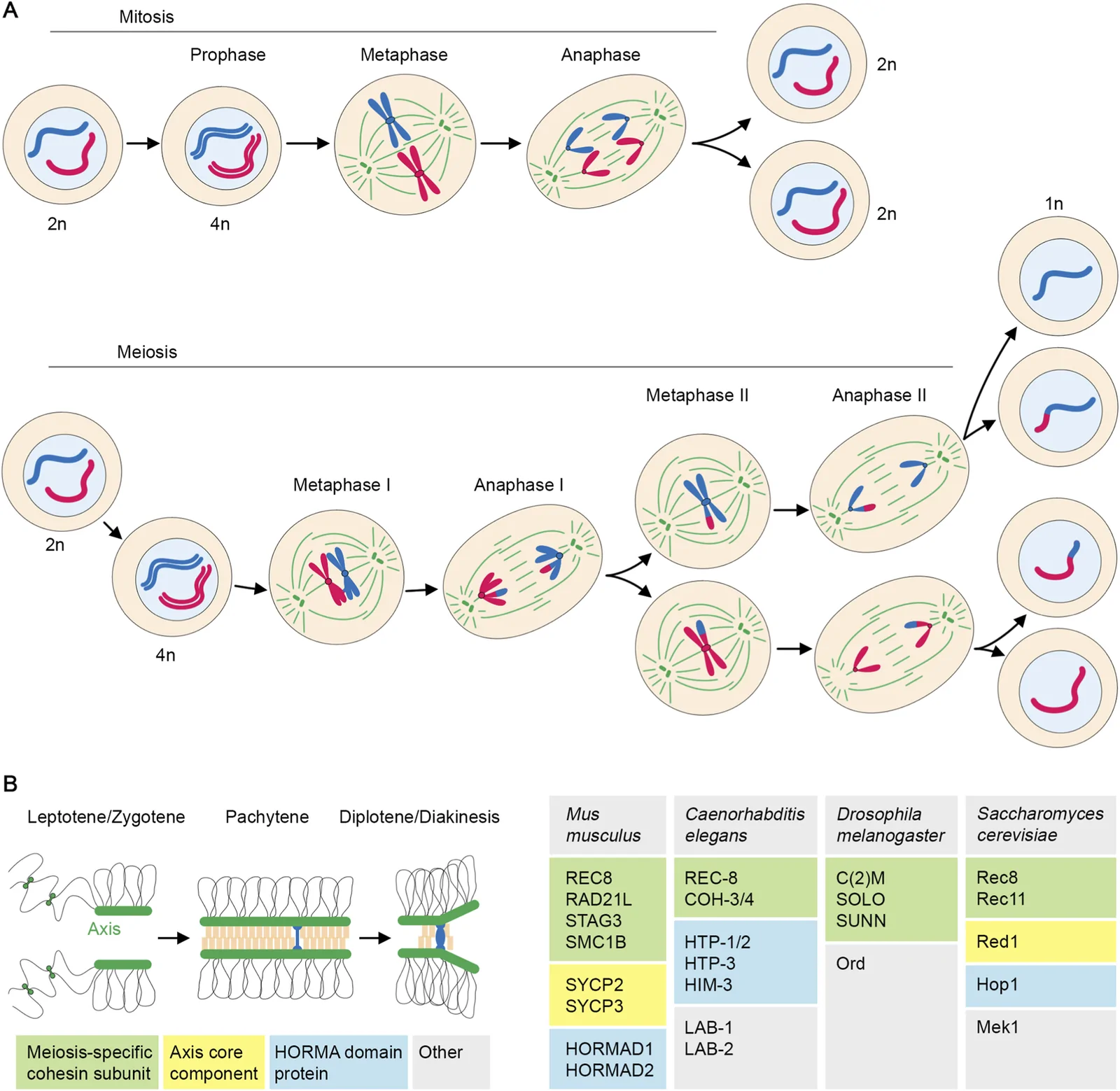
Overview of cell division programs and meiotic chromosome loop-axis organization. **(A)** Comparison of chromosome segregation behaviors during mitotic and meiotic cell divisions. Only one pair of homologous chromosomes is illustrated. **(B)** Meiotic chromosome loop-axis organization and axis components in mouse, worm, fly, and yeast. Ubiquitously expressed cohesin subunits that form cohesin rings and constitute major components of the chromosome axis are not shown. Blue bridges between homolog axes represent recombination intermediates and crossovers.

Meiosis is a specialized cellular program that produces haploid gametes from diploid germ cells while generating genetic diversity through homologous recombination and chromosome reshuffling. Unlike mitosis, in which a single round of DNA replication is followed by segregation of sister chromatids, meiosis consists of two successive chromosome segregation events (meiosis I and meiosis II) following only one round of replication. Meiosis I is reductional, separating homologous chromosomes, whereas meiosis II resembles mitosis and separates sister chromatids (Figure 1A). Accurate segregation during meiosis I requires stable physical connections between homologs, which are primarily provided by crossovers (COs) and sister chromatid cohesion.

To establish these connections, chromosomes undergo an extended prophase during which homologs pair, synapse, and recombine, ultimately forming chiasmata that ensure accurate segregation at the first meiotic division.

Meiotic chromosomes face several unique challenges. Homologs must locate and recognize one another within the confined nuclear volume despite initially lacking stable physical connections and being spatially separated. Their interactions must then be selectively stabilized through pairing, synapsis, and recombination while preventing inappropriate associations between nonhomologous chromosomes. At the same time, CO formation must be tightly controlled to ensure at least one CO per homolog pair while limiting CO number and shaping their distribution along the chromosomes. Finally, local recombination events must be coordinated with chromosome-wide structural remodeling to generate chromosome configurations capable of supporting faithful segregation.

In this review, we examine the mechanisms that drive and regulate chromosome motion during meiotic prophase and discuss how these dynamics help overcome the major chromosomal challenges of meiosis. It is important to note that the meiotic program varies among organisms, and many of its key components are not conserved at the protein sequence level despite performing functionally analogous roles. Accordingly, we discuss these mechanisms from a comparative perspective, with a particular focus on several widely used model organisms: mouse, *Drosophila*, *Caenorhabditis elegans*, and yeast.

## Loop-axis organization sets the framework for meiotic events

Throughout prophase, each homolog is organized as an array of sister chromatid loops emanating from a shared axial core (Figure 1B). This conserved loop-axis architecture is assembled from cohesins, HORMA (Hop1, Rev7, Mad2) domain proteins, condensins, topoisomerase II, and other chromosome-associated factors (Grey and de Massy, 2021; Gyuricza et al., 2016; Hong et al., 2019; Kohler et al., 2017; Sakuno et al., 2022; Ur and Corbett, 2021; Wang et al., 2025b; Woglar et al., 2020; Zhang F. G. et al., 2021). Meiotic isoforms of cohesins and HORMA proteins are the most consistently conserved elements, while axis length and assembly are dynamically regulated by cohesin/condensin turnover, post-translational modifications, ATP-dependent chromatin remodeling, and transcriptional activity (Challa et al., 2016; Heldrich et al., 2022; Ur and Corbett, 2021; Wang et al., 2025b; Wang et al., 2021; Yang et al., 2022).

The loop-axis organization provides the structural foundation for nearly all major meiotic chromosome events. Programmed DNA double-strand breaks (DSBs) are generated in chromatin loops that are tethered to the chromosome axis, where recombination intermediates are further processed (Ito and Shinohara, 2023). The axis also promotes the homolog bias of meiotic recombination and coordinates local DNA repair events with chromosome-wide organization (Ito and Shinohara, 2023; Zickler and Kleckner, 2023). During homolog pairing, chromosome axes become aligned, and subsequent assembly of the synaptonemal complex (SC) between homologous axes stabilizes synapsis bringing and maintaining an interaxis distance of ∼100 nm, with chromatin loops projecting outward from each axis. As prophase progresses, chromosomes undergo extensive remodeling, including SC disassembly and chromosome compaction, ultimately leaving homologs connected only at CO sites.

Thus, the meiotic chromosome axis serves not merely as a structural scaffold but as an organizational platform that integrates chromosome movement, homolog interactions, recombination, and chromosome remodeling throughout meiotic prophase.

## The meiotic bouquet and bouquet-like configurations

**FIGURE 2:**
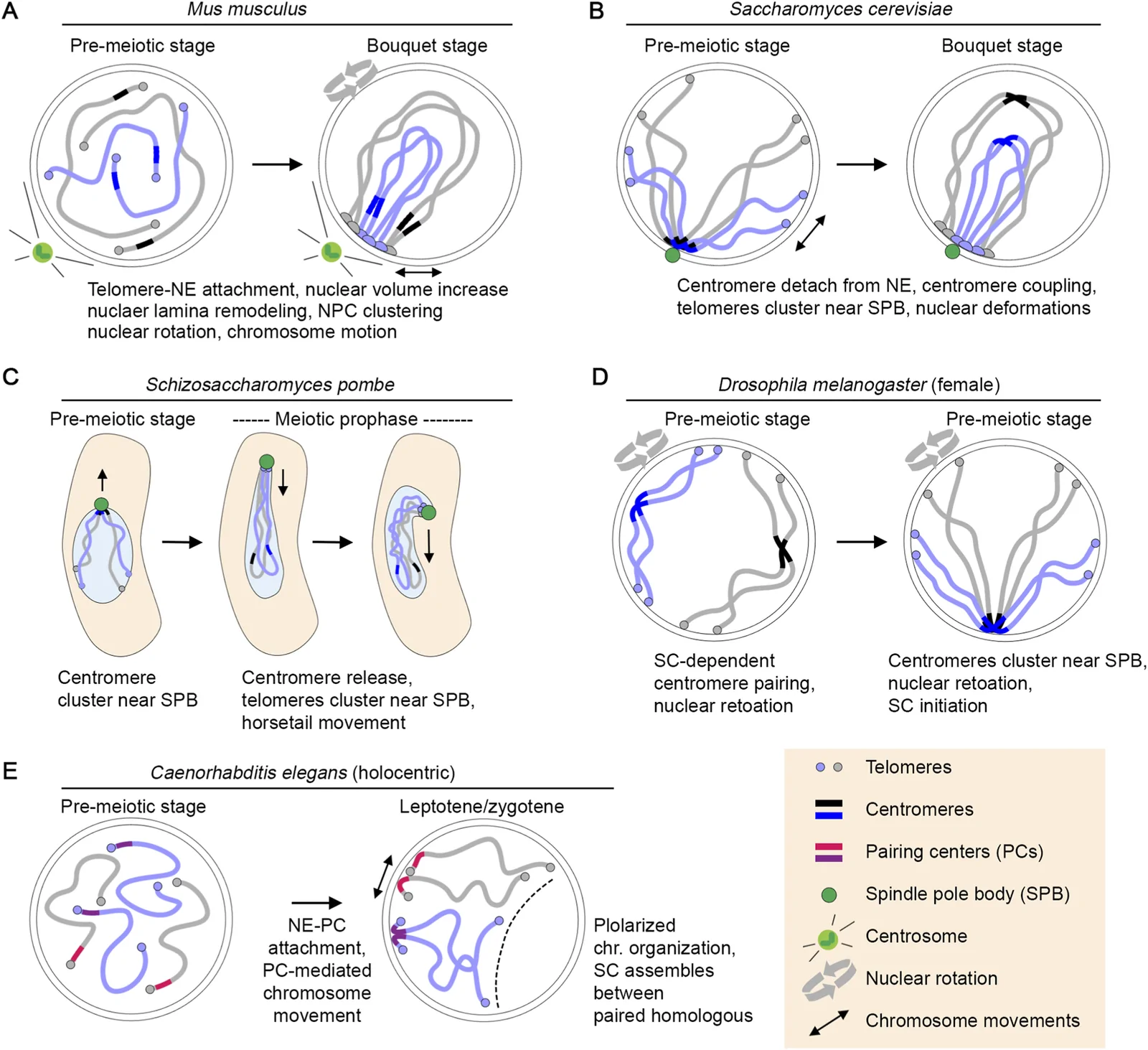
Meiotic bouquet configuration. **(A,B)** Classical meiotic bouquet in mouse **(A)** and budding yeast **(B)**. Circular arrows indicate nuclear rotation, and double-headed arrows indicate chromosome movement. **(C–E)** Bouquet analogs in fission yeast **(C)**, fly **(D)**, and worm **(E)**. In fission yeast, the nucleus undergoes elongation and whole-nucleus movement. In female flies, homologous pairing is established during the pre-meiotic stage. In *Caenorhabditis elegans*, subtelomeric pairing centers mediate chromosome clustering.

One of the most conspicuous chromosome configurations during meiotic prophase is the bouquet, in which chromosome ends attach to the nuclear envelope (NE) and cluster within a restricted region of the nuclear periphery. First described nearly a century ago, bouquet formation is widely conserved across kingdoms and is considered a hallmark of early meiotic prophase (Cromer et al., 2024; Scherthan, 2001; Zickler and Kleckner, 2016) (Figures 2A–C). Although telomeres mediate bouquet formation in most organisms, analogous chromosome clustering can be achieved through other specialized chromosomal domains, such as centromeres in *Drosophila melanogaster* and pairing centers in *C. elegans* (Figures 2D,E).

**FIGURE 3:**
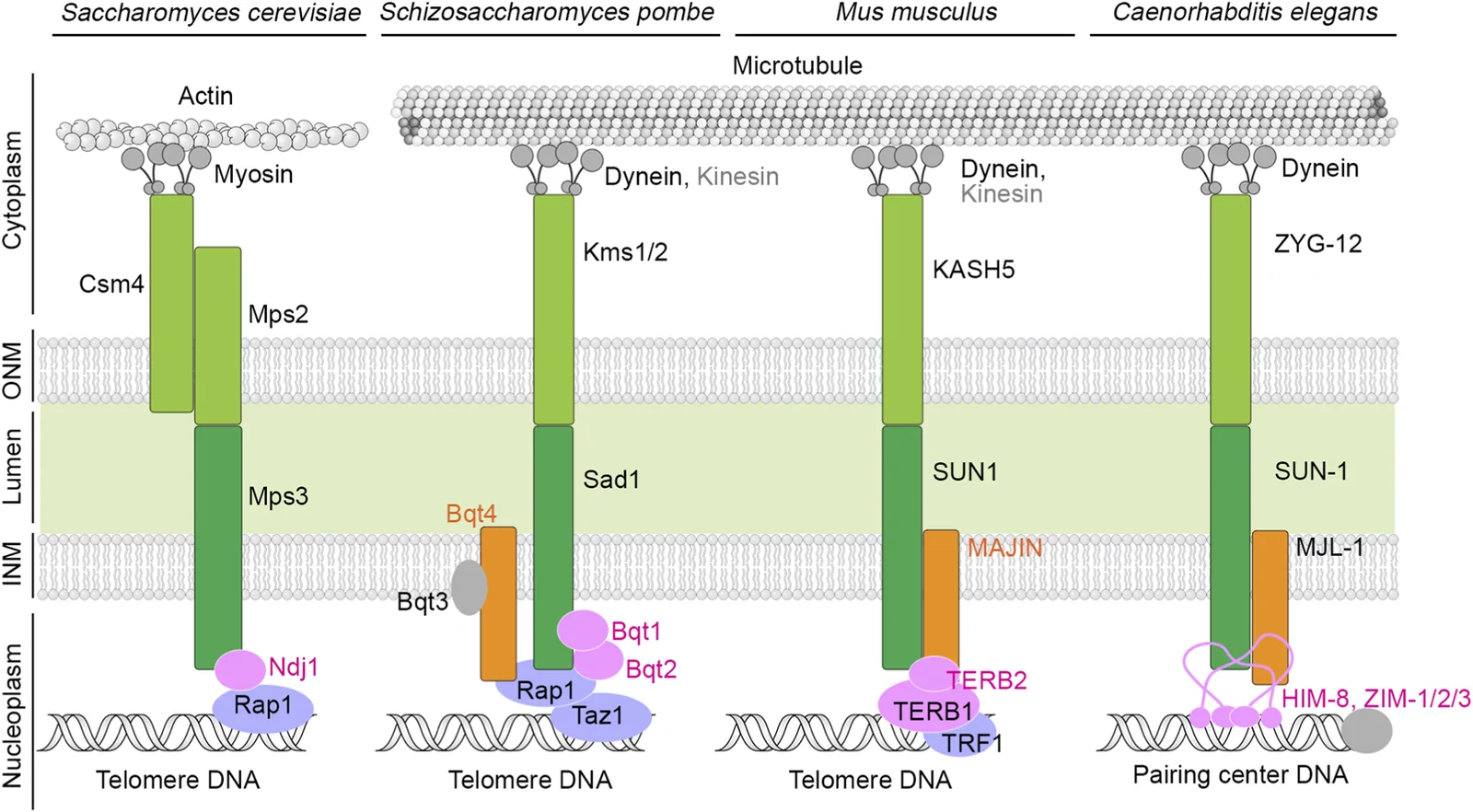
Transmission of cytoplasmic forces through the nuclear envelope. Summary of the structural components mediating connections between nuclear chromosomes and the cytoplasmic cytoskeleton in budding yeast, fission yeast, mouse, and worm. For the LINC complex, higher-order oligomeric assemblies beyond homotrimers may exist (McGillivary et al., 2023), although the precise protein stoichiometries remain to be determined. The contribution of kinesin to chromosome movement may be mediated indirectly through its effects on microtubule stability. Additional cofactors and adaptors of cytoskeletal motors (e.g., dynactin) that may contribute to chromosome movements, as well as kinases regulating chromosome end–nuclear envelope attachment, are not illustrated. ONM, outer nuclear membrane; NE, nuclear envelope; INM, inner nuclear membrane.

Chromosome-end clustering is tightly associated with active chromosome motion. Chromosome ends anchored to the NE are mechanically coupled to cytoskeletal motors through the linker of nucleoskeleton and cytoskeleton (LINC) complex. Spanning the double-membrane NE, the LINC complex comprises SUN (Sad1 and UNC-84 homology) domain proteins in the inner nuclear membrane (INM) and KASH (Klarsicht, ANC-1, and Syne homology) domain proteins in the outer nuclear membrane (ONM), thereby transmitting cytoskeletal forces from the cytoplasm to chromosomes (Chikashige et al., 2006; Shibuya, 2025; Zetka et al., 2020) (Figure 3). Bouquet formation is therefore thought to emerge from the collective behavior of actively moving chromosome ends. However, the mechanisms that drive tight chromosome-end clustering and the subsequent dissolution of the bouquet during pachytene remain poorly understood. Notably, bouquet formation is accompanied by extensive reorganization of the NE, including the exclusion of nuclear pore complexes from telomere-rich regions (Zickler and Kleckner, 1998), suggesting that large-scale membrane remodeling may contribute to chromosome-end clustering.

## Specialized chromosomal domains mediating chromosome motion

### Telomeres

Telomeres are the most widespread chromosomal domains responsible for coupling meiotic chromosomes to the NE. In addition to protecting chromosome ends, telomeres provide attachment sites for meiosis-specific protein complexes that connect chromosomes to LINC complexes. Through these connections, cytoplasmic forces generated by microtubule- or actin-based motors can be transmitted to chromosomes, driving the characteristic chromosome movements observed during early meiotic prophase.

Telomeres possess several properties that may facilitate their meiotic functions. They form specialized higher-order structures through interactions between telomeric DNA and shelterin proteins, and increasing evidence suggests that telomeric components can undergo phase separation, a fundamental mechanism of biomolecular self-organization that promotes the formation of membraneless condensates and recruitment of specific factors (Jack et al., 2022; Lee et al., 2024; Zhang H. et al., 2020). Such self-organizing behaviors may facilitate chromosome-end protection, kinase recruitment, the dynamic assembly of chromosome-NE attachment complexes, telomere clustering, and bouquet formation. In mice, TRF1 contributes to both telomere protection and NE tethering during meiosis, acting with factors such as Speedy A and CDK2 to prevent telomere fusion (Wang et al., 2018). In plants, telomeres and subtelomeres are highly variable in sequence, which may facilitate homolog recognition and pairing at the onset of meiosis, as suggested in barley (Serrano-Leon et al., 2023). Elucidating the higher-order organization and phase-separation behavior of telomeres will be key to understanding their meiotic functions.

### Pairing centers in *Caenorhabditis elegans*

Unlike most organisms, *C. elegans* does not rely on telomeres to mediate chromosome motion. Instead, each chromosome contains a specialized pairing center (PC) located near chromosome ends. PCs recruit a family of zinc-finger proteins (HIM-8 and ZIM-1/2/3) that link chromosomes to LINC complexes at the NE, creating dedicated sites for force transmission (Baudrimont et al., 2010; Phillips and Dernburg, 2006). PCs are composed of repetitive DNA sequences that selectively recruit distinct zinc-finger proteins. Each chromosome recruits only one PC-binding protein to promote homolog recognition and pairing: chromosomes II and III recruit ZIM-1, chromosome V recruits ZIM-2, chromosomes I and IV recruit ZIM-3, and the X chromosome recruits HIM-8. High-resolution imaging and biochemical analyses suggest that PC-binding proteins assemble DNA-containing compartments that are spatially offset from the chromosome axis and serve as sites for both NE attachment and homology recognition (Wang et al., 2025c).

### Centromeres

In several species, centromeres contribute to chromosome dynamics during meiotic prophase. Before homolog pairing, centromeres often undergo transient clustering or coupling, generating higher-order chromosome configurations that shape nuclear organization. In *Drosophila*, centromeres serve as the primary chromosome domains that connect meiotic chromosomes to the NE and mediate chromosome motion (Fellmeth and McKim, 2022; Rubin et al., 2022).

Beyond their role in kinetochore assembly, centromeres possess intrinsic self-association properties arising from their specialized nucleosome composition and heterochromatic environment (Bell et al., 2025; Brahmachari et al., 2022; Trivedi et al., 2019). These properties may promote centromere clustering and nuclear compartmentalization and may facilitate coordinated chromosome behavior during early meiotic prophase.

## The machinery driving chromosome motion

In most organisms examined to date, chromosome ends attach to the NE throughout the pairing period, providing a platform for cytoskeleton-driven chromosome movements. Tracking chromosome ends using chromosome markers or LINC complex components has revealed average movement velocities of ∼50–120 nm/s during the active chromosome movement stage (Link and Jantsch, 2019; Nozaki et al., 2021; Nozaki et al., 2024; Shibuya, 2025; Wynne et al., 2012; Xie et al., 2025; Zhang R. et al., 2024). In some species, cytoskeletal forces also drive global rotation of the meiotic nucleus (Link and Jantsch, 2019).

### NE association of chromosome ends

In fission yeast, telomeres in vegetative cells are steadily attached to the INM via shelterin protein Rap1 and INM protein Bqt3/4 (Chikashige et al., 2009). After meiotic entry, the meiosis-specific telomere proteins Bqt1/2 are recruited to the telomere-NE interface by Rap1 (Chikashige et al., 2006). This, in turn, induces the recruitment of the LINC complexes composed of the SUN and KASH proteins Sad1 and Kms1/2, respectively. In budding yeast, the functional homolog of Bqt1/2, Ndj1, mediates the connection between telomere and the LINC complex, which is composed of the SUN-domain protein Mps3 and two KASH-like proteins Mps2 and Csm4. Through this linkage, actin-based myosin motors transmit cytoskeletal forces to drive chromosome movements (Conrad et al., 2008; Lee et al., 2020).

The interaction network is better characterized and more complex in mammals than in yeast (Shibuya, 2025). In mice, the meiosis-specific telomere repeat-binding bouquet formation protein 1 (TERB1), TERB2, and the INM protein membrane-anchored junction protein (MAJIN) connect telomeres to the NE. In this connection, TERB1 interacts with the shelterin protein TERF1, while TERB2 bridges TERB1 to MAJIN (Shibuya et al., 2015; Shibuya et al., 2014a). This TERB1/2-MAJIN complex recruits the LINC complex composed of SUN1 and KASH5, and both TERB1 and MAJIN interact with the nucleoplasmic domain of SUN1 (Shibuya et al., 2014a; Wang et al., 2020). Mutations in TERB1/2-MAJIN and LINC complex proteins all cause severe defects in pairing and recombination, leading to infertility in both sexes in mice (Shibuya, 2025). In humans, accumulating evidence suggests that mutations in SUN1, SUN5, KASH5, and other telomere-attachment factors are associated with infertility (Bentebbal et al., 2021; Hou et al., 2023; Meng et al., 2023; Xiang et al., 2022; Zhang D. et al., 2021; Zhu et al., 2016).

Notably, although the complexes mediating telomere-NE attachments, Bqt1/2 in fission yeast, Ndj1 in budding yeast, and TERB1/2-MAJIN in mammals, are functionally conserved, these components show little sequence similarity. However, TERB1/2 and MAJIN are conserved in most metazoan.

In *C. elegans*, which lacks TERB1/2 homologs, the core components mediating PC-NE attachment include the INM proteins SUN-1, MJL-1 (a functional homolog of mammalian MAJIN), and the PC-binding proteins (HIM-8, ZIM-1/2/3) (Kim et al., 2023; Zetka et al., 2020). Interestingly, the nucleoplasmic regions of SUN-1 and MJL-1, together with the PC-binding proteins, are largely intrinsically disordered. Evidence suggests that PC-NE attachment involves phase separation, which may facilitate kinase recruitment and homolog recognition (Wang et al., 2025c).

Recruitment of kinases to chromosome-NE attachment sites is likely conserved across phyla. In *C. elegans*, PLK-1/2 and CHK-2 localize to the PC-NE interface and are required for pairing (Harper et al., 2011; Kim et al., 2015; Labella et al., 2011). Similarly, in mice, cyclin-dependent kinase 2 (Cdk2) and its noncanonical activator Speedy A are recruited to the telomere-NE interface (Tu et al., 2017). However, how kinase activity promotes PC-NE attachment and pairing remains not fully understood.

### Interactions within the NE lumen

The SUN- and KASH-domain proteins interact immediately below the ONM (Sosa et al., 2012). In mammals, there are five SUN proteins (SUN1-5) and six KASH proteins (nesprin/SYNE-1–4, KASH5, and LRMP/IRAG2) that are expressed and undergo extensive alternative splicing in a tissue-specific manner (Jahed et al., 2016; Razafsky and Hodzic, 2009). KASH proteins vary at both their cytoplasmic N-termini and KASH domains: nesprin-1/2 bind actin through their N-terminal tandem calponin homology domains and interact with kinesin light chain through a C-terminal W-acidic motif, enabling cross-linking of the actin and microtubule cytoskeletons (Luxton et al., 2025; Sahabandu et al., 2025); nesprin-3 binds plectin to couple the LINC complex to intermediate filaments, whereas nesprin-4 binds kinesin light chain through a C-terminal W-acidic motif (Luxton et al., 2025) and the meiosis-specific KASH5 binds dynein (Zi-Yi et al., 2024). Notably, the KASH domains of nesprin-4 and KASH5 exhibit substantial sequence diversity (Horn et al., 2013).

Structural studies show that SUN2 forms trimers that bind three KASH peptides, yielding a hexameric complex (Sosa et al., 2012). A mesh-like network of SUN-KASH interactions, involving both head-on and lateral associations, has been proposed (Gurusaran and Davies, 2021; Jahed et al., 2021; Jahed et al., 2018). Several in-cell measurements have provided insights into LINC complex assembly *in vivo*, suggesting that distinct assembly states, including heteromeric complexes, may form to support diverse mechanotransduction processes and undergo dynamic remodeling (Hennen et al., 2019; Hennen et al., 2020; Hennen et al., 2018). However, how various oligomeric state accommodate specific functions, and how tissue-specific isoforms and alternative splicing regulate the stability and specialization of these transmembrane interactions, remain important questions for future investigation.

### Cytoplasmic sources of forces

The cytoskeletal forces driving chromosome movements depend on the motor proteins associated with KASH-domain proteins. Accordingly, either microtubules or actin filaments, together with their respective motors, generate the forces required for chromosome movement. In *C. elegans*, dynein-dependent processive chromosome motions promote homologous pairing (Wynne et al., 2012). In *Arabidopsis*, the KASH-domain protein SINE3 recruits the kinesin PSS1 to the NE, facilitating bouquet formation and rapid chromosome movements, thereby promoting synapsis and proper CO distribution (Cai et al., 2025; Duroc et al., 2014). In budding yeast, rapid chromosome movement rely on actin filament and myosin (Lee et al., 2020). In fission yeast and mouse, both dynein and kinesins (Klp6/5 in fission yeast and KIF5B in mice) have been implicated in driving rapid chromosome movements, although kinesins may function indirectly by regulating microtubule dynamics (Ditamo et al., 2025; Shibuya, 2025).

As LINC complex clustering is triggered by the NE attachment of chromosome ends, the corresponding clustering of cytoskeletal motors is likely to follow. These motor assemblies may capture cytoskeletal filaments surrounding the nucleus. Because cytoskeletal filaments are intrinsically polarized, clustered motors could become coordinated in their directionality, thereby generating sufficient force to drive chromosome movements. Whether this mechanism operates *in vivo* remains to be determined. Notably, the cytoplasmic N-terminus of KASH5 directly binds the dynein light chain and functions as an activating adaptor (Agrawal et al., 2022; Garner et al., 2023).

## Regulation of chromosome motion

Meiotic chromosome movements require not only the assembly of chromosome-NE attachment and force-transmission machinery, but also its precise regulation in space and time. Chromosome dynamics are controlled by multiple mechanisms, including meiosis-specific expression of isoforms, kinase-dependent regulation of chromosome-NE attachment, modulation of nuclear membrane and lamina biophysical properties, coordination with meiotic progression, and physical constraints imposed by chromosome architecture and nuclear organization.

### Sex-specific regulation

Meiotic chromosome dynamics often differ markedly between the sexes (Hua and Liu, 2021; Ishiguro, 2022; Rubin et al., 2022). These differences can include the duration of meiotic prophase, chromosome length, chromosome movement speed, and even the requirement for recombination and synapsis. Consequently, mutations in the same gene can have distinct effects on male and female fertility.

In *Drosophila*, chromosome movements are much slower in males than in females (Rubin et al., 2022). However, extending prophase is sufficient to rescue pairing-defective mutants and promote proper centromere pairing (Rubin et al., 2022). In *C. elegans*, meiotic progression is faster in males than in females (Jaramillo-Lambert et al., 2007). Defects in chromosome axis assembly are more severe in males, whereas delayed meiotic progression in aged females can partially rescue synapsis defects (Liu et al., 2026; Wang et al., 2025b). The molecular basis of these sex-specific regulation remains largely unknown.

### Kinase regulation of NE attachment

Kinase-mediated phosphorylation of proteins has emerged as an essential mechanism regulating telomere-NE attachment. In mice, CDK2 and its noncanonical activator SPDYA are recruited to telomeres throughout meiotic prophase (Mikolcevic et al., 2016). Homozygous mutations in these genes disrupt telomere attachment to the NE and cause infertility in both sexes (Mikolcevic et al., 2016; Viera et al., 2015). *In vitro* and genetic analyses indicate that telomeric localization of the CDK2-SPDYA complex and SUN1 is interdependent. Specifically, CDK2 localization to telomeres depends on SUN1. Biochemical studies further show that the N-terminal domain of SUN1 interacts with SPDYA and is phosphorylated by the CDK2-SPDYA complex (Mikolcevic et al., 2016; Wang et al., 2020). Consistently, a SUN1 point mutation (W151R) that disrupts SPDYA binding phenocopies *Sun1* and *Spdya* knockout mice (Chen et al., 2021). In *C. elegans*, the kinases CHK-2 and PLK-2 are recruited to PCs and are essential for homolog pairing, although the relevant phosphorylation targets required for this process remain to be identified (Kim et al., 2015; Wang et al., 2025c).

### Nuclear membrane fluidity

At the environment in which the LINC complexes are embedded, NE membrane composition, which directly influences membrane fluidity and mechanical properties, may play an important role in shaping meiotic chromosome dynamics. Membrane fluidity can be enhanced by fatty acid elongases, which extend fatty acid carbon chains, and fatty acid desaturases, which introduce additional double bonds into fatty acyl chains (de Mendoza and Pilon, 2019; Pilon and Ruiz, 2023). Homozygous deletion mutants of the elongase elongation of very long-chain fatty acids protein 2 (ELOVL2), the ceramide synthase CerS3, or the fatty acid desaturase FADS2 causes male infertility, with the last also causing female infertility (Rabionet et al., 2008; Stoffel et al., 2020; Zadravec et al., 2011).

The conserved membrane fluidity regulator adiponectin receptor 2 (AdipoR2) upregulate the expression of ELOVL2, thereby promoting the synthesis of very-long-chain polyunsaturated fatty acids (VLC-PUFAs). *AdipoR2* knockout (KO) spermatocytes show abnormally membrane packing, as measured by Laurdan dye staining, accompanied by defects in homolog pairing and recombination and resulting in male infertility (Zhang J. et al., 2024). Notably, *AdipoR2* deletion mutation does not affect telomere-NE attachment, and dynein-dependent movement forces cause NE curvature and subsequent invagination without driving telomere movements. How nuclear membrane fluidity is regulated between mammalian sexes, across different organisms, and its impact on chromosomes dynamics remain to be investigated.

### Nuclear lamina dynamics

The nuclear lamina consists of a polymeric protein network that supports the INM as a structure scaffold (Gruenbaum and Foisner, 2015). This network imposes mechanical constraints on NE attachment and chromosome movement, and its dynamics instability are likely enhanced during early meiotic prophase (Link et al., 2018; Shibuya, 2025). Two major strategies appear to underlie this remodeling: incorporation of meiosis-specific lamin isoforms and local regulation through kinase phosphorylation.

Based on their biochemical properties and the types of networks they form, lamins are classified into A- and B-type families, with most invertebrates expressing a single lamin that is more closely related to B-type lamins (Gruenbaum and Foisner, 2015). Mammalian cells express both A- and B-type lamins with multiple isoforms. A-type lamins (lamin A, lamin C, lamin C2, and lamin AΔ10) are encoded by a single gene (*LMNA* in humans), whereas B-type lamins (lamin B1, lamin B2, and lamin B3) are encoded by two genes, *LMNB1* and *LMNB2*, which have overlapping yet distinct cellular and developmental functions (Gruenbaum and Foisner, 2015). Notably, lamin C2 and lamin B3, encoded by *LMNA* and *LMNB2*, respectively, are testis-specific isoforms (Burke and Stewart, 2013). Isoform-specific homozygous deletion of lamin C2 in mice causes synapsis defects and male-specific infertility (Link et al., 2013). Incorporation of lamin C2 may fluidize the nuclear lamina, thereby facilitating telomere-driven chromosome movements during meiosis.

Kinase-mediated phosphorylation plays an essential role in promoting chromosome-NE attachment. In *C. elegans*, phosphorylation of the sole lamin protein LMN-1 by CHK-2 and PLK-2 at PC-NE attachment sites enhances nuclear lamina fluidity (Link et al., 2018). Kinase-dependent regulation of nuclear lamina during meiotic chromosome-NE attachment and movement may share mechanistic principles with lamin remodeling during mitosis, where cellular kinases, including protein kinase A, protein kinase C, MAP kinases, and cyclin dependent kinases, regulate lamin dynamics (Liu S. et al., 2025; Torvaldson et al., 2015; Wesley et al., 2024). Similar principles may also underlie localized lamin disassembly during herpesvirus infection, which is driven by viral kinase activity (Morrison and DeLassus, 2011).

Lamins directly interact with chromatin and have profound effects on chromatin organization and gene regulation in interphase cells (Dechat et al., 2009; Wang B. et al., 2025). During early meiotic prophase, these lamin-chromatin interactions are likely to be transiently relaxed to permit efficient chromosome movements and homolog pairing. Consistent with this idea, in *Arabidopsis*, the meiosis-specific SCF(RMF) E3 ubiquitin ligase disrupts the nuclear lamina to release somatic constraints and enable homolog pairing (Yuan et al., 2025). Similarly, in *C. elegans*, BAF-1-VRK-1 mediated release of meiotic chromosomes from the nuclear periphery is important for gamete genome integrity (Paouneskou et al., 2025). Notably, in late prophase oocytes, the lamin network is re-established to help stabilize the nucleus against mechanical stress (Liu et al., 2023).

### Coordination of chromosome motion with meiotic progression

Chromosome motion is tightly coordinated with meiotic progression. In *C. elegans*, chromosome clustering is mechanistically linked to pairing. Mutations in chromosome axis components prevent both clustering and pairing (Couteau et al., 2004; Martinez-Perez and Villeneuve, 2005; Severson et al., 2009), suggesting that an intact axis is required for active chromosome movement. In *htp-1* mutants, extensive nonhomologous synapsis occurs (Couteau and Zetka, 2005; Martinez-Perez and Villeneuve, 2005), supporting a role for chromosome motion in preventing inappropriate chromosome interactions. Thus, chromosome motion appears functionally coupled to intact axis assembly, thereby minimizing chromatin entanglements and DNA damage upon meiotic entry.

The signals that terminate chromosome movement remain not fully understood. In recombination mutants of mice and yeast, bouquet disassembly is delayed (Conrad et al., 2008; Moiseeva et al., 2017; Shibuya et al., 2014a), suggesting that specific recombination intermediates or completion of DNA repair promote bouquet exit. Similarly, synapsis defects in *C. elegans* prolong the stage characterized by chromosome clustering and active movement. Proposed mechanisms include mechanical forces generated during SC morphogenesis and signaling pathways linked to synapsis progression (MacQueen et al., 2002). Consistent with this idea, experimental SC removal can reactivate chromosome movement before late pachytene (Castellano-Pozo et al., 2020), suggesting that asynapsis sustains the active motion state. In pachytene, impaired axis integrity appears to slow or inhibit chromosome movements (Zhang R. et al., 2024).

The CHK-2 kinase has emerged as a key coordinator of chromosome organization and movement in *C. elegans* (Kim et al., 2015), although the underlying signaling mechanisms remain to be elucidated.

### Balance of chromosome size, nuclear space, and driving forces

Efficient homolog pairing likely requires coordination among cytoplasmic driving forces, chromosome size, and nuclear volume. Excessive forces can generate large-scale nuclear fluctuations, whereas moderate forces are more effective at driving directed chromosome movements. In *C. elegans*, GRAS-1, the homolog of mammalian GRASP/Tamalin and CYTIP, localizes to the NE and restrains dynein-dependent chromosome movements, thereby coordinating homolog search with SC assembly (Martinez-Garcia et al., 2023).

**FIGURE 4:**
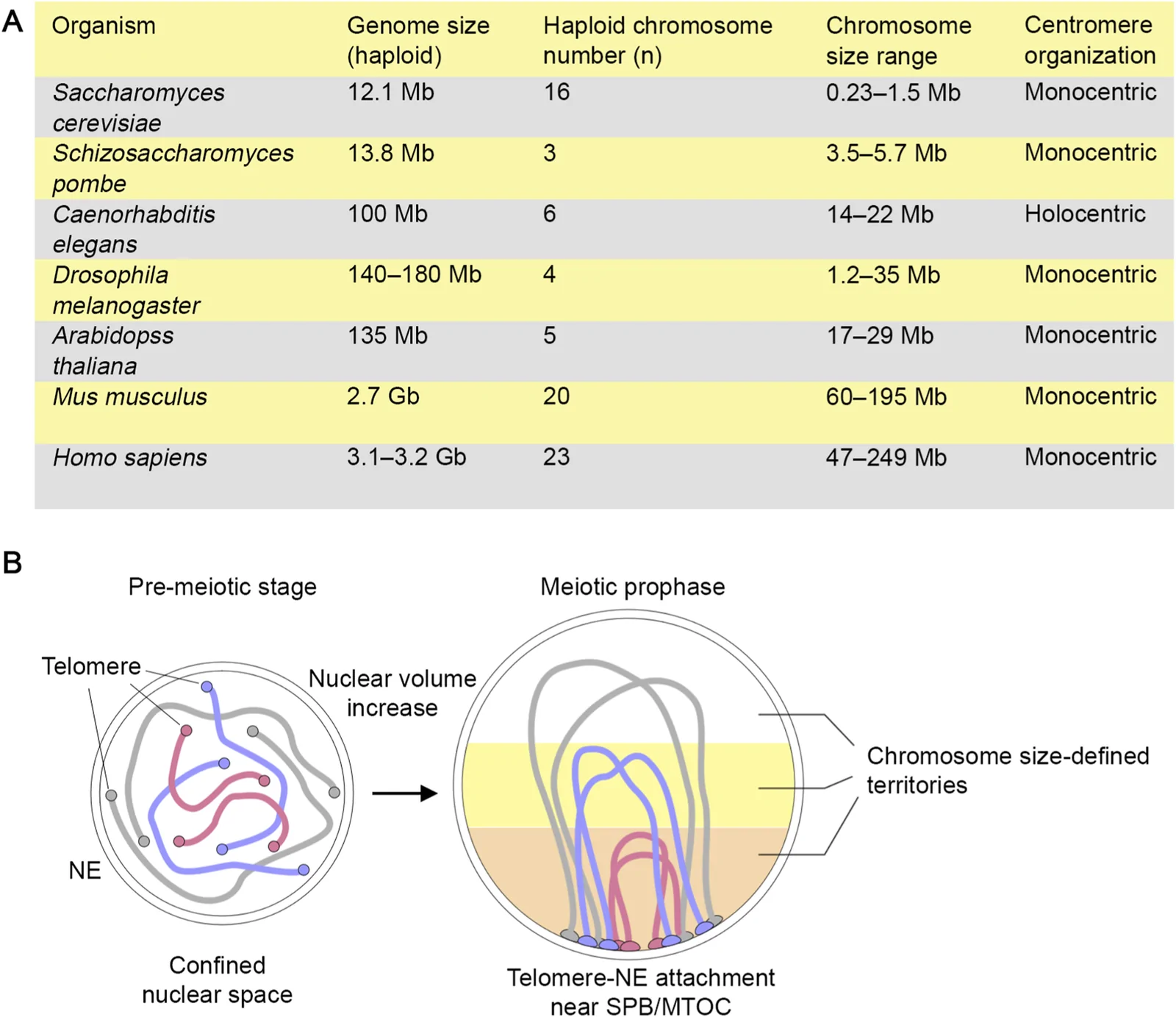
Impact of chromosome size on homologous pairing. **(A)** Comparative genomic and chromosomal features of representative organisms used in meiotic studies. **(B)** Telomere-NE attachment and clustering help organize chromosomes into distinct nuclear territories, thereby promoting pairing efficiency. This process is facilitated by an increase in nuclear volume.

A nearly universal feature of meiosis is nuclear expansion during homolog pairing and synapsis. Increased nuclear volume likely accommodates chromosome alignment and facilitates the resolution of interlocks generated by active chromosome movements. Efficient pairing also appears to depend on chromosomes falling within an optimal size range rather than being uniform in length (Li et al., 2011) (Figure 4). Overly long chromosomes are difficult to align within the confined nuclear space, whereas modest size differences may promote automatic co-alignment during the bouquet stage. Consistent with this idea, chromosome fusions that increase chromosome length cause meiotic defects in fission yeast (Jiang et al., 2025).

Active chromosome movement is also energy-intensive. Consistent with this, impairment of mitochondrial respiration disrupts SUN-1 aggregate dynamics in *C. elegans* (Labrador et al., 2013).

## Biological functions of chromosome motion

Chromosome end-led movements during meiotic prophase are among the most conserved features of meiosis. They are essential not only for canonical pairing, but also in organisms such as *Drosophila* males, which lack both recombination and the SC (Rubin et al., 2022). Functionally, chromosome end-led coordinated movements likely serve several interrelated roles, including promoting the formation of the chromosome bouquet, facilitating homologous pairing, and resolving chromosomal entanglements. In some organisms, chromosome movements persist after synapsis is complete and they may also have postsynapsis functions.

### Homolog pairing

Homologous pairing represents one of the central challenges of meiosis. Because homologs initially occupy distinct positions within the nucleus, mechanisms are required to increase encounter frequency while maintaining pairing fidelity. Chromosome end attachment to the NE contributes to this process by reducing the homology search from three-dimensional nuclear space to a two-dimensional surface. Dynamic chromosome movements further enhance pairing by continuously reshaping chromosome configurations and generating new opportunities for homologous loci to encounter one another (Zickler and Kleckner, 2023).

In fission yeast, all telomeres cluster at the spindle pole body (the fungal equivalent of the centrosome) and the nucleus undergo rapid back-and-forth sweeping movement, known as “horsetail” oscillations, along the longitudinal axis of the cell (Chacon et al., 2016) (Figure 2C). During each oscillation cycle, chromosomes become transiently aligned, placing corresponding chromosomal regions in similar spatial orientations relative to the telomere cluster. Repeated changes in movement direction continually reorganize these relationships, thereby increasing the probability that previously unpaired homologs encounter one another. Consistent with this model, recombination-independent pairing is established during early oscillations, whereas recombination-mediated interactions emerge later (Ding et al., 2004).

Similar principles appear to operate in other organisms despite substantial differences in chromosome organization. In budding yeast and *C. elegans*, chromosome ends form dynamic groups that move in distinct directions over time (Koszul et al., 2008; Woglar and Jantsch, 2014). Successive episodes of coordinated movement involving different chromosome subsets could progressively propagate pairing-promoting effects throughout the genome. Similarly, global nuclear rotation, which affects all chromosome ends simultaneously, could extend these effects from chromosome ends into chromosome interiors.

**FIGURE 5:**
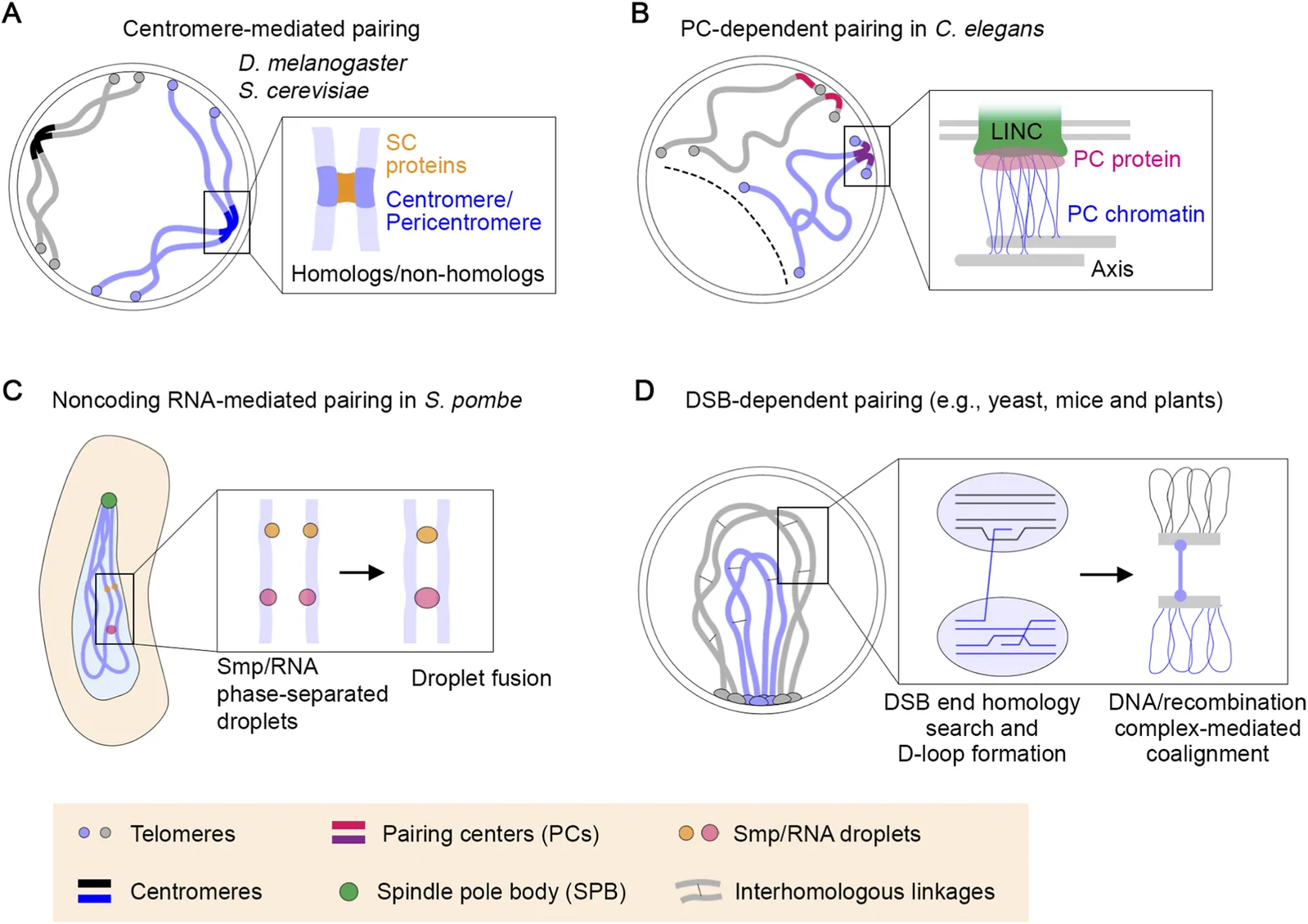
Mechanisms of DSB-independent and DSB-dependent pairing. **(A)** Centromere-mediated pairing in *Drosophila* and budding yeast. SC components are required for this type of pairing, which precedes or occurs independently of DSB formation. **(B)** Pairing center (PC)-mediated pairing in *Caenorhabditis elegans*. PC-binding proteins form aggregates and interact with the LINC complex beneath the nuclear envelope. **(C)** Noncoding RNA-mediated pairing in *S. pombe*, which also precedes meiotic recombination. Pairing involves the fusion of droplets formed by noncoding RNAs and Smp proteins. **(D)** DSB-dependent pairing occurs in many organisms after DSB formation. This process involves DSB end separation and D-loop formation, followed by physical interactions between recombination complexes and the chromosome axis, generating stable homologous linkages.

In many organisms, stable pairing and synapsis can be achieved without recombination, including *D. melanogaster*, *Bombyx mori* females, and *C. elegans* (Figures 5A–C). In these species, SC assembly can precede, bypass, or occur independently of recombination (Dernburg et al., 1998; Fellmeth and McKim, 2022; Rubin et al., 2022), although the underlying mechanisms appear to vary. In fission yeast, genome-wide pairing relies on a noncoding RNA-mediated phase-separation process (Ding et al., 2019; Ding et al., 2024). In *C. elegans*, PCs promote homolog recognition within phase-separated compartments beneath the NE, potentially involving direct DNA-DNA interactions (Wang et al., 2025c). In *Drosophila*, non-recombinational pairing is a hallmark of somatic cells, is lost in the germline, and is re-established during early meiosis (Joyce et al., 2013). In *Drosophila* male meiosis I, homologs are stably conjoined by DNA-binding protein SNM, which is cleaved by separase at anaphase I (Kabakci et al., 2022).

Recombination-independent homolog associations also occur in budding yeast and mice, although recombination is ultimately required for complete synapsis. In budding yeast, early prophase centromere coupling occurs in a homology-independent manner, followed by homologous centromere pairing; SC proteins are required and promote proper segregation of achiasmate chromosomes (Goldman and Lichten, 1996; Kurdzo et al., 2017). In mice, meiosis-specific cohesins mediate homolog recognition or association upon meiotic entry, prior to axis assembly and DSB formation (Ishiguro et al., 2014).

In species that rely on recombination for pairing, full alignment can only take place after DSBs are properly formed and processed (Zickler and Kleckner, 2015). DNA-level homology recognition generates nascent displacement loops (D-loops), three-stranded DNA structures formed by strand invasion, which establish the initial physical links between homologs in an “ends-apart” geometry. These recombination-mediated interactions associate with chromosome axes, forming paired recombination complexes distributed along homologs, each corresponding to a DSB end (Figure 5D). Together, these interactions align homolog axes at an inter-axis distance of ∼400 nm and promote SC assembly (Storlazzi et al., 2010).

### Synapsis

**FIGURE 6:**
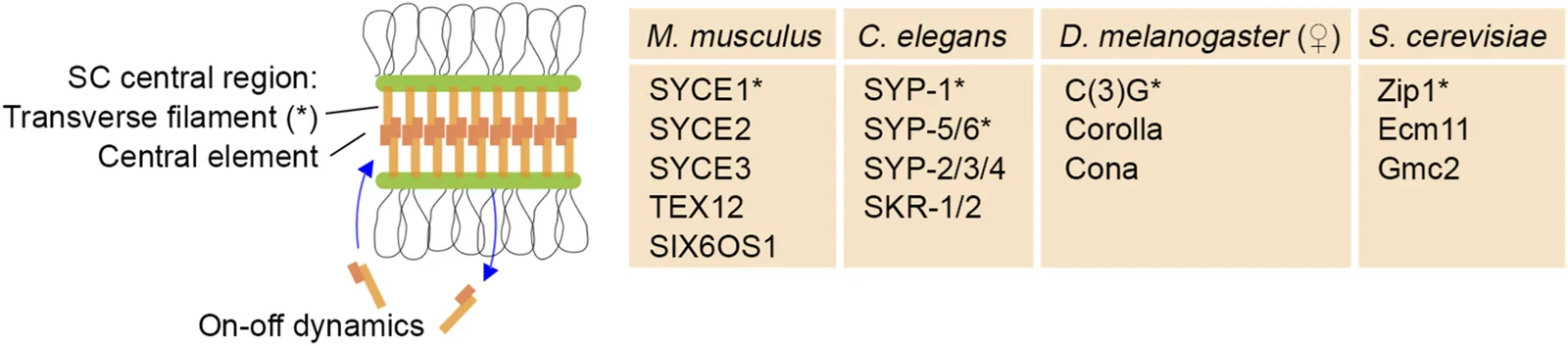
SC structure and central region components. SC organization and components in mouse, worm, fly, and budding yeast. The SC central region usually consists of transverse filaments (*) and central element proteins. In *C. elegans*, the non-canonical SC structural components SKR-1/2 are also present. SC components are dynamic within the structure.

Following homolog alignment, chromosome movements continue to influence synapsis, a process in which the conserved tripartite SC structure assembled between the chromosome axes and maintains them ∼100 nm apart (Figure 6). Canonical SC proteins are enriched in coiled-coil domains and assemble into subcomplexes that bridge homologous chromosome axes (Gao and Colaiacovo, 2018; Zhang F. G. et al., 2021). Mutations in SC components are associated with human infertility (Zhang et al., 2022).

The SC is now recognized as a dynamic structure whose assembly and axis association are mediated by multivalent weak interactions (Gordon et al., 2025; Wang et al., 2024; Zhang et al., 2023; Zhang Z. et al., 2020). Although it is a prominent cytological hallmark of homolog pairing, the SC does not mediate homolog recognition or initial alignment. Consistent with its intrinsic self-assembly capacity, SC components can assemble under noncanonical conditions, including between nonhomologous chromosomes, along a folded chromosome, or as polycomplexes (Hughes and Hawley, 2020; Liu et al., 2021; Nora et al., 2013). SC nucleation is generally the rate-limiting step of synapsis; once nucleated between coaligned axes, SC elongation proceeds rapidly along the chromosomes (Rog and Dernburg, 2015).

The intrinsic assembly properties of the SC suggest that chromosome movements likely promote synapsis through two complementary mechanisms. First, they facilitate axis straightening and close alignment, creating optimal spatial conditions for SC nucleation and subsequent elongation. In organisms where SC nucleation occurs at recombination intermediates, chromosome movements may likewise promote SC extension by optimizing axis coalignment. Second, chromosome movements help prevent inappropriate SC assembly between nonhomologous chromosomes. Because chromosomes are confined within a limited nuclear volume and SC proteins readily self-assemble. Dynamic chromosome movements destabilize incorrect chromosome associations, thereby enhancing synapsis fidelity. Consistent with this model, in *C. elegans*, the SC fails to assemble efficiently in the absence of chromosome movements, even when PC-end pairing has been established (Sato et al., 2009).

### Resolution of topological chromosome interlocks

**FIGURE 7:**
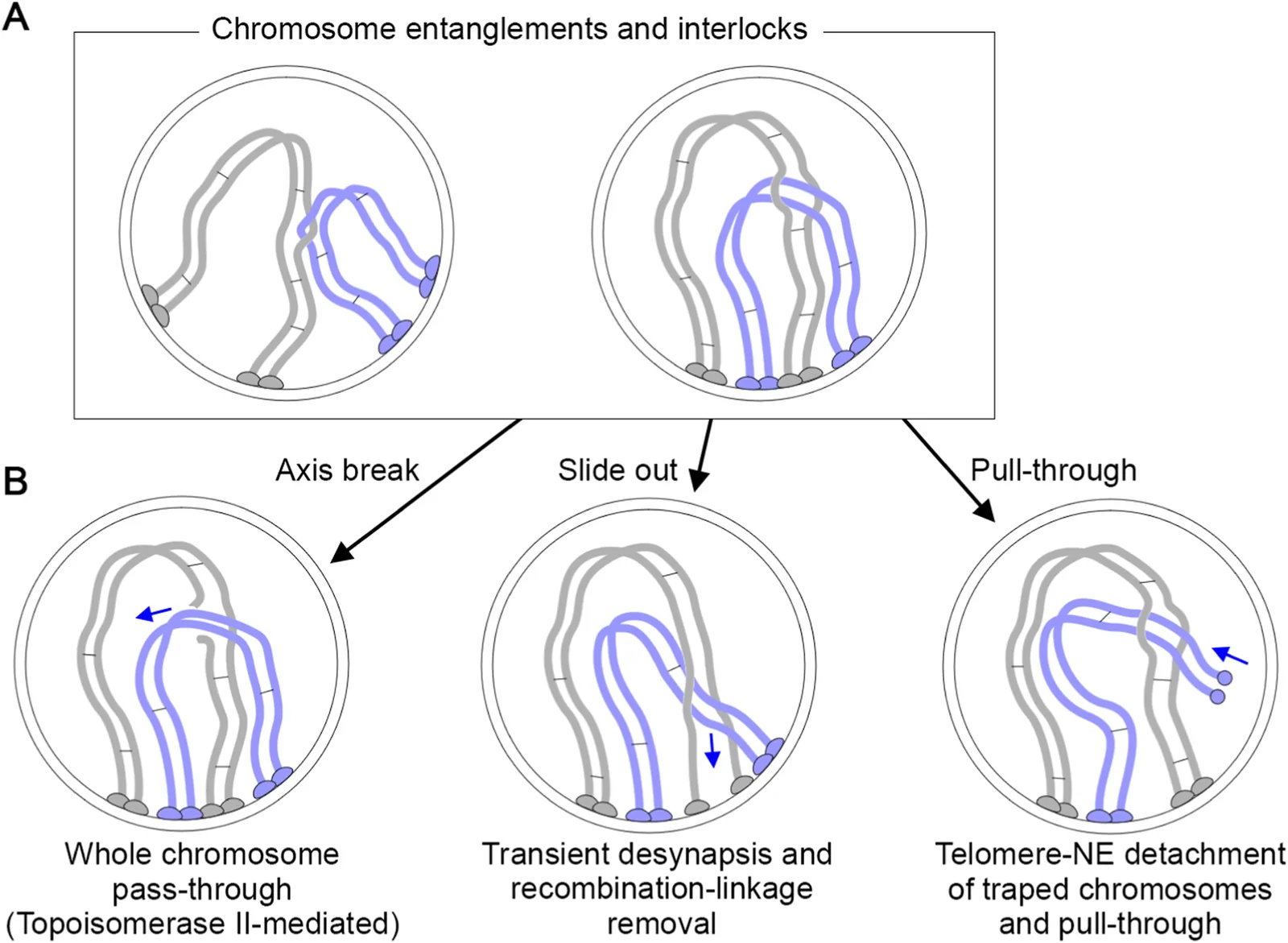
Chromosome interlocks and pathways of resolution. **(A)** Schematic representation of chromosome entanglements and interlocks during early meiotic prophase. Telomeres are attached to the NE, and recombination intermediates may form between homologs. **(B)** Three pathways of interlock resolution: axis breakage of the constraining chromosomes, potentially involving topoisomerase II; slide-out of the trapped chromosome, requiring partial desynapsis of the constraining chromosomes; and the pull-through pathway, involving detachment of the trapped chromosome telomeres from the NE.

Interlocks arise when a chromosome or chromosome pair becomes topologically trapped, resulting in SC formation on both sides of the trapped region (Zickler and Kleckner, 1999) (Figure 7A). Similar topological entanglements can also occur before SC assembly. However, such configurations are absent by mid-pachytene (Storlazzi et al., 2010), suggesting that they are actively removed.

Resolving such topological constraints requires relative displacement between the trapped and constraining chromosomes. Based on cytological observations from wild-type meiosis and meiotic mutants across multiple organisms (Martinez-Garcia et al., 2018; Rasmussen, 1986; Storlazzi et al., 2010; von Wettstein et al., 1984), three general mechanisms have been proposed: (1) breakage of the constraining chromosome to allow passage; (2) outward movement of the trapped chromosome toward chromosome ends; and (3) detachment from the NE, enabling escape through the constraining region (Olaya et al., 2024; Zickler and Kleckner, 2023) (Figure 7B). Chromosome movements are most directly compatible with models involving active displacement of trapped chromosomes.

The dynamic nature of the SC may facilitate interlock resolution through local disassembly and reassembly. However, recombination-dependent linkages between homologs create additional constraints by generating stable chromosome connections. Several pathways may help alleviate these barriers. CO interference can direct recombination intermediates toward non-CO outcomes, thereby reducing homolog-bridging connections, particularly when homolog alignment and interference occur concurrently, as observed in autotetraploid *Arabidopsis* (Morgan et al., 2021). DNA damage-response pathways may also process stalled recombination intermediates into NCOs or sister-chromatid repair products; consistent with this, loss of DNA mismatch repair protein Mlh1 increases interlock frequency (Storlazzi et al., 2010). In addition, topoisomerases and helicases likely contribute to interlock resolution. Topo II loss increases interlocks in *Arabidopsis* (Martinez-Garcia et al., 2018), suggesting roles in strand passage, decatenation, or resolution of recombination-associated DNA linkages.

### Potential landscape regulation of recombination and CO distribution

The period of rapid chromosome movement coincides with recombination and CO designation, suggesting that chromosome dynamics may influence both recombination outcomes and large-scale distribution of COs along chromosomes (CO landscape). In *C. elegans*, chromosome movements have been implicated in DSB repair pathway choice (Lawrence et al., 2016).

In most organisms, COs are distributed nonuniformly along chromosomes (Pazhayam et al., 2021). This pattern reflects not only CO interference but also global regulation of CO designation. Beyond their potential role in interlock resolution, where chromosome displacement may channel some recombination intermediates into NCOs, chromosome movements may influence CO distribution through spatiotemporal regulation of meiotic progression. In barley, wheat, and mouse, DSB formation, early recombination intermediates, and synapsis initiate preferentially near chromosome ends and subsequently progress inward (Higgins et al., 2022; Higgins et al., 2014; Osman et al., 2021; Pratto et al., 2021). COs preferentially arise in regions that lead this progression, whereas more delayed interstitial regions tend to produce NCOs.

Consistent with a role for chromosome dynamics in CO patterning, disruption of telomere-NE attachment in *Arabidopsis* abolishes rapid chromosome movements, impairs synapsis, and causes CO clustering (Cai et al., 2025). Likewise, in *C. elegans*, COs occur preferentially near chromosome ends, particularly on autosomes (Saito and Colaiacovo, 2017). These observations suggest that chromosome movements may help establish spatiotemporal differences in recombination progression, thereby shaping CO distribution along chromosomes.

### Post-synapsis functions of chromosome motion

Although chromosome movements are traditionally viewed as mechanisms that promote homolog pairing and synapsis, accumulating evidence suggests that their functions extend beyond these early meiotic events. In mice, *C. elegans*, and budding yeast, chromosome movements persist into pachytene, after pairing and synapsis are largely complete (Conrad et al., 2008; Koszul et al., 2008; Lee et al., 2015; Shibuya et al., 2014b).

In mouse spermatocytes, XY chromosomes undergo DSB-dependent pairing within the pseudoautosomal region (PAR) during late zygonema. Nonhomologous synapsis occurs between the entire or nearly entire Y-axis and the X-axis, which is transient and subsequently resolved through nonhomologous desynapsis, restricting the SC to the PAR by mid-pachytene (Acquaviva et al., 2020; Liu D. et al., 2025). Pachytene-specific knockout of *SpdyA*, a component stabilizing telomere-NE attachment, results in the failure of XY nonhomologous desynapsis (Liu D. et al., 2025), supporting a model in which telomere-led chromosome movements facilitate desynapsis.

Chromosomes undergo extensive structural reorganization during the pachytene-to-diplotene transition (Martinez-Perez et al., 2008; Zickler and Kleckner, 2023), but whether active forces contribute to this remodeling remains unclear. In *C. elegans*, chromosome ends detach from the NE and the nuclear lamina becomes reinforced during late pachytene (Liu et al., 2023; Wang et al., 2025c). Although these observations argue against a major role for trans-NE forces at this stage, it may also reflect a requirement for a mechanically strengthened lamina to withstand increasing cytoplasmic forces. Whether intranuclear force-generating mechanisms, such as actin- and myosin-based systems, continue to drive chromosome movements during late prophase, and what functions these movements might serve, remain important open questions.

## Future perspectives

Despite substantial advances in understanding meiotic chromosome dynamics, many fundamental questions remain unresolved. A major challenge is to elucidate the organization and biophysical properties of the specialized chromosome domains that mediate NE attachment. How telomeres, PCs, and centromeres are assembled into NE-associated structures, how their composition and organization change during meiotic progression and how these changes are regulated remain poorly understood. In particular, the mechanisms by which centromeres establish NE attachment and mediate chromosome coupling in species that rely on centromere-led movements are largely unknown.

The mechanisms governing chromosome-NE interactions also require further investigation. The forces that direct chromosome ends or centromeres toward the NE at meiotic entry, and the potential existence of surveillance pathways that monitor attachment integrity remain open questions. How chromosome-axis architecture influences chromosome mobility is another important area for future study, as increasing evidence suggests that chromosome structure and motion are tightly coupled.

On the cytoplasmic side of the NE, the force-generating machinery remains incompletely characterized. Future work should define how microtubules, actin filaments, motor proteins, and their regulatory networks are organized during meiosis and whether they are specialized for chromosome movement. The contribution of classical microtubule-organizing centers to meiotic microtubule organization, as well as the mechanisms underlying zygotene cilium formation in zebrafish and mice (Lopez-Jimenez et al., 2022; Mytlis et al., 2022) and its functional significance, are particularly intriguing questions.

Perhaps the most important unresolved issue is the biological significance of chromosome movement itself. While chromosome dynamics clearly promote homolog pairing and synapsis (Zickler and Kleckner, 2023), their contributions to recombination, CO designation, CO distribution, and chromosome remodeling during later stages of prophase remain poorly understood.

Future studies should also explore how chromosome dynamics vary across biological contexts, including sex-specific meiotic programs, chromosome size-dependent effects, polyploidy, and aging. Advances in high-resolution four-dimensional live-cell imaging, exemplified by recent approaches developed in budding yeast (Nozaki et al., 2024), should enable direct visualization of chromosome trajectories and interactions throughout meiotic prophase, providing unprecedented insights into the mechanisms and functions of meiotic chromosome dynamics.

Exploring meiotic chromosome dynamics from quantitative and biophysical perspectives should provide important insights into their underlying principles and regulatory mechanisms. Relevant biophysical parameters include chromosome stiffness and elasticity, nuclear confinement, force measurements, and single-molecule analyses. Combined with quantitative biophysical measurements and computational modeling, such as polymer simulations (Schalbetter et al., 2019), these approaches should enable a more predictive understanding of how chromosome movements promote homolog recognition, recombination, and ultimately faithful chromosome segregation.
